# Lead-free perovskite KCsSnI_1.7_Cl_1.3_ material exhibiting superior photocatalytic antimicrobial activity

**DOI:** 10.1038/s41598-025-17357-8

**Published:** 2025-09-01

**Authors:** Ahmed M. Mahmoud, Canan Can, Mohamed Rabia

**Affiliations:** 1 Department of Botany and Microbiology, Faculty of Science, Beni-Suef University, Beni-Suef, 62511 Egypt; 2 Faculty of Arts and Sciences, Division of Molecular Biology, Department of Biology, Gaziantep University, Gaziantep, Turkey; 3 Nanomaterials Science Research Laboratory, Chemistry Department, Faculty of Science, Beni-Suef University, Beni-Suef, Egypt

**Keywords:** KCsSnI_1.7_Cl_1.3_, Perovskite, Antimicrobial, Bacteria, Material, Biophysics, Biotechnology, Nanoscience and technology

## Abstract

The development of environmentally friendly and highly efficient materials is critical for next-generation antibacterial and optoelectronic applications. In this study, we present the successful synthesis of a novel lead-free perovskite, KCsSnI_1.7_Cl_1.3_, via a rapid and scalable chemical bath deposition method at 150 °C for just 5 min. The resulting film features well-defined orthorhombic, pyramid-like crystals with uniform grain sizes (800–1000 nm) and a compact, pinhole-free morphology. Remarkably, the material exhibits strong optical absorption up to 800 nm, positioning it as a promising candidate for hot electron generation and light-driven applications. KCsSnI_1.7_Cl_1.3_ also demonstrated outstanding antibacterial performance, showing broad-spectrum activity against multiple bacterial strains. The highest inhibition was recorded against *Staphylococcus aureus*, with inhibition zones increasing from 17 mm in the dark to 35 mm under UV illumination—highlighting its efficient photocatalytic response. The antibacterial effect followed the order: *S. aureus* > *B. subtilis* > *E. coli* > *Salmonella* sp., and was significantly enhanced with increasing concentrations (100–400 ppm). No inhibition was observed in control wells, confirming the selective activity of the material. With its lead-free composition, strong light-harvesting ability, and exceptional antibacterial properties, KCsSnI_1.7_Cl_1.3_ emerges as a highly promising material for future applications in antibacterial coatings, water purification systems, and advanced optoelectronic devices. Its green synthesis, scalability, and multifunctionality offer a sustainable pathway toward real-world deployment in environmental and biomedical fields.

**Supplementary Information:**

The online version contains supplementary material available at 10.1038/s41598-025-17357-8.

## Introduction

Microbial infections pose significant biological threats to humans, animals, and plants. These infections are caused by various bacteria or viruses^1^. Many individuals are afflicted by severe and life-threatening diseases resulting from bacterial infections, such as those caused by *Escherichia coli* (*E. coli*) and *Staphylococcus aureus* (*S. aureus*)^2–4^. Extensive research is being conducted to protect biological systems from microbial attacks, including efforts to safeguard plants, animals, and humans^5^.

The development of antimicrobial materials is a rapidly advancing area of research. Various studies have explored using noble metals, such as silver, for their antibacterial properties against Gram-negative and Gram-positive bacteria. Silver is known for its effective antibacterial activity^6,7^. Additionally, gold exhibits some antimicrobial properties against certain bacteria, although its effectiveness is significantly reduced against *E. coli*^8^. However, noble metals are limited due to their high cost and varying effectiveness against different bacterial strains. Furthermore, these metals can accumulate in the human body without being metabolized or degraded, potentially leading to health issues.

Recent research in antimicrobial materials has increasingly focused on overcoming the limitations of conventional agents by developing cost-effective, environmentally friendly alternatives with high efficacy and minimal harm to human health. Among the various candidates, perovskite nanomaterials have emerged as a rapidly advancing class, particularly for antimicrobial and photocatalytic applications.

While traditional lead-based perovskites are well-known for their outstanding optoelectronic properties, their widespread use is constrained by the toxicity and environmental hazards associated with lead. To mitigate these concerns, lead-free perovskites incorporating benign elements such as cesium and tin have gained considerable attention. These materials offer a compelling combination of strong optical absorption, tunable band gaps, and significantly reduced toxicity^9–11^.

Lead-free perovskites have demonstrated excellent photocatalytic performance under visible light, enabling efficient degradation of organic pollutants and conversion of carbon dioxide^12^. Their high absorption coefficients, adjustable energy levels, and efficient charge transport characteristics also render them ideal for optoelectronic applications such as solar energy harvesting and light-emitting devices^13–15^. Additionally, their stability under ambient conditions enhances their suitability for real-world, sustainable technologies^15,16^.

Beyond their optoelectronic advantages, lead-free perovskites have shown potent antimicrobial activity, attributed to their ability to disrupt microbial membranes and induce oxidative stress through reactive oxygen species (ROS) generation. These mechanisms lead to cellular damage—including protein denaturation and DNA fragmentation—ultimately resulting in microbial cell death^17,18^. This multi-targeted approach not only enhances their effectiveness against a wide spectrum of pathogens but also reduces the likelihood of resistance development, presenting a valuable alternative to traditional antibiotics^19^. Moreover, by fine-tuning the material composition and structure, the antimicrobial performance of these perovskites can be further optimized^20,21^.

Lead-free perovskites also hold promise for integration into coatings, water purification systems, and antimicrobial composites, providing eco-friendly and scalable solutions for controlling pathogenic threats in medical, environmental, and industrial settings^22–24^. Their multifunctionality positions them as key materials to address pressing global challenges related to public health and environmental sustainability.

In this study, we report the synthesis and characterization of a novel lead-free perovskite, KCsSnI_1.7_Cl_1.3_, and evaluate its structural, optical, and antibacterial properties, emphasizing its potential as a dual-function material for light-driven and antimicrobial applications.

## Materials and methods

### Materials

Cesium chloride (CsCl, 99.9%, Sigma Aldrich, Japan), KI (99.9%, Pio-chem, Egypt), tin chloride (SnCl_2_, 999%, Sigma Aldrich, USA), and N, N-dimethylformamide (DMF, 99.9% Sigma Aldrich, USA).

### Preparation of the lead-free perovskite material KCsSnI_1.7_Cl_1.3_

The KCsSnI_1.7_Cl_1.3_ lead-free perovskite nanomaterial was synthesized using the chemical bath deposition method. A mixture of CsCl, KI, and SnCl_2_ in a ratio of 1:3:1 was heated at 80 °C for 1 h. Subsequently, the temperature was raised to 150 °C for 5 min, resulting in the formation of a yellow-orange colored KCsSnI_1.7_Cl_1.3_ perovskite material. through this preparation, the *N*,*N*-dimethylformamide is used as a solvent.

### Characterization and analyses

The chemical structure of the material was confirmed through X-ray diffraction (XRD) analysis using the PANalytical Pro (Holland) model, Cu Kα radiation (λ = 1.5406 Å), and X-ray photoelectron spectroscopy (XPS) using the K-ALPHA (USA) model, Al Kα (hν = 1486.6 eV). Optical properties were analyzed using a UV/Vis spectrophotometer (Perkin Elmer, USA). The morphology of the material was examined with a scanning electron microscope (SEM), model ZEISS (Gemini), and a transmission electron microscope (TEM), model JEOL JEM-2100.

### Antibacterial activity tests

The antibacterial activity of the lead-free perovskite material KCsSnI_1.7_Cl_1.3_ is assessed in vitro using the agar well diffusion method^25^. This technique involved measuring the growth inhibition zones on Muller Hinton Agar (MHA). The human pathogens tested included Gram-negative bacteria (*Salmonella* sp. and *E. coli*) and Gram-positive bacteria (*S. aureus* and *B. subtilis*). To evaluate the photocatalytic activity of the nanomaterial, the pathogens were incubated under UV light (UV lamp with a wavelength of 254 nm and an intensity of 1.5 mW/cm^2^), and the increase in inhibition zone diameters was monitored. In this procedure, 100 µl of a 24-hour-old Muller Hinton broth culture (5 × 10^6^ CFU) of each pathogen was added to 100 ml of MHA at 45 °C and pH 7, then poured into three Petri dishes. Once solidified, wells (5 mm) were created using a sterilized borer, and 50 µl of different nanomaterial concentrations (100, 200, and 400 ppm) were aseptically introduced into the wells. As a control, one well in each plate was filled with the supernatant from each pathogen. The plates were incubated at 37 °C for 24 h, after which the diameters of the inhibition zones (in millimeters) around the wells were measured and recorded.

## Results and discussion

### Characterization

**Fig. 1:**
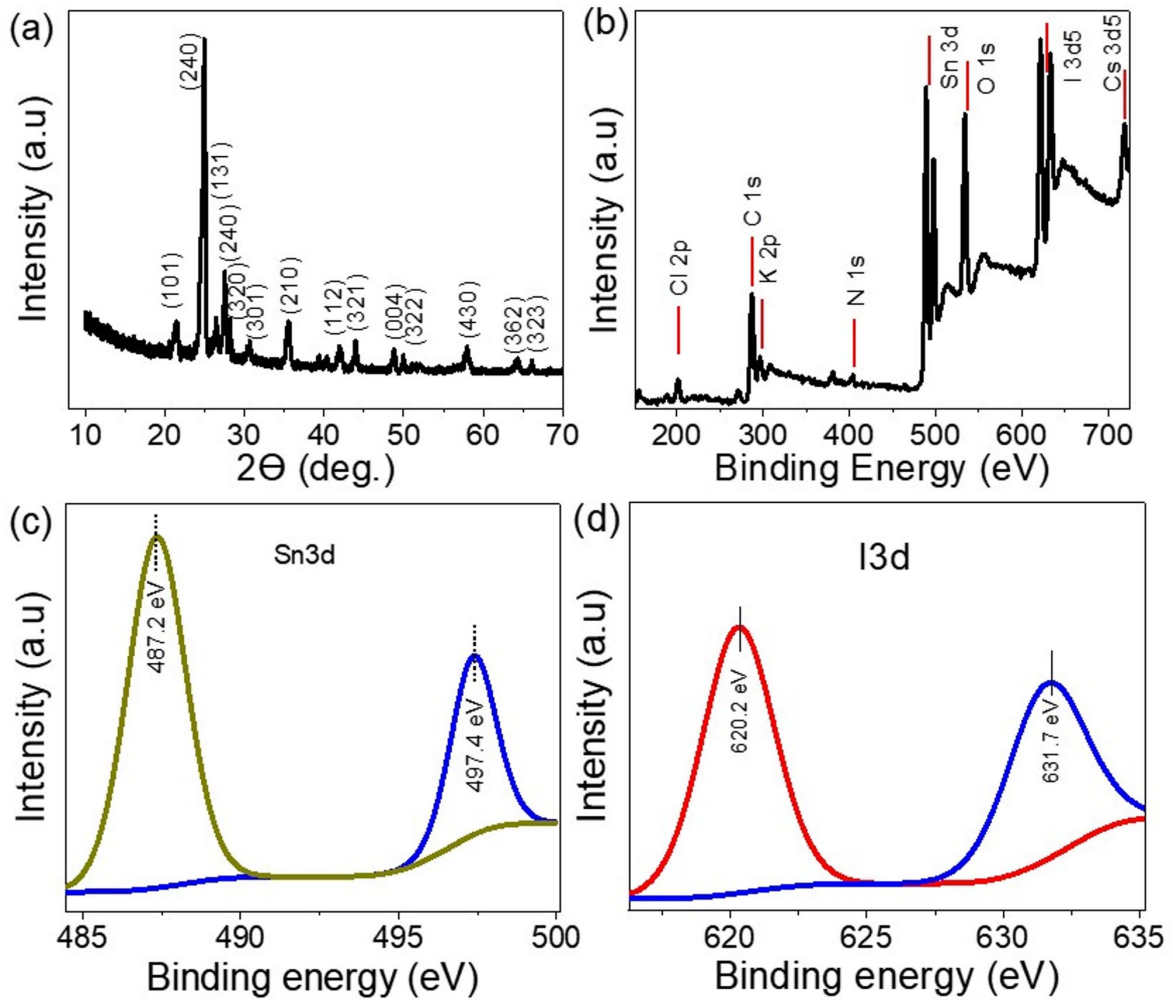
(**a**) XRD pattern and (**b**–**d**) XPS total survey and spectra for Sn and I elements, respectively, of the lead-free perovskite KCsSnI_1.7_Cl_1.3_ material.

The XRD of the prepared lead-free perovskite KCsSnI_1.7_Cl_1.3_ nanomaterials is shown in Fig. 1a, from this figure, the prepared material has 14 peaks that characterize it, these peaks located at 21.55, 25.0, 26.44, 27.54, 28.18, 30.66, 35.55, 41.94, 43.87, 48.77, 49.9, 58.0, 64.43, and 66.11o for the growth direction (101), (240), (131), (240), (320), (301), (210), (112), (321), (004), (322), (430), (362), and (323), respectively, card # 75-0376^26^. these peaks are matched well with the previous study^27^. The great intensity peaks confirm the formation of high-intensity perovskite crystals. Scherrer’s formula, Eq. 1^28,29^ can be used to determine the crystal size (D) of lead-free perovskite KCsSnI_1.7_Cl_1.3_, the determination depends on some parameters; W, k, λ, and θ, which represents full-width half maximum, dimensionless factor, X-ray wavelength, and Bragg angle. From this equation, the crystal size of the perovskite material at highest peak (25.0) is 31 nm.

1 $${\text{D = 0}}{\text{.9}}\lambda {\text{ / W cos}}\theta$$

The XPS survey for the lead-free perovskite materials is shown in Fig. 1b, from this figure, the characteristics elements for the perovskite appear well, in which Cl 2p, K 2p, Sn 3d, I 3d5, Cs 3d5 are located at 202.7, 296.4, 487.2, 620.1, and 718.8 eV, respectively. the spectra for the Sn and I is estimated through 3d as estimated in Fig. 1c,d, respectively.

In addition to that, there are additional elements that appear: C 1s, N 1s, and O 1s. These elements are related to the solvent of the perovskite materials DMF, in which these peaks are located at 287.1, 404.0, and 534.8 eV, respectively. These solvent behaviors are well matched with previous literature^30^. The presence of the perovskite anions Cl^–^ and I^–^ constitutes 5.0% and 6.23% of the sample, respectively. The cations K, Cs, and Sn present with the percentages 2.46, 2.42, and 6.92%, respectively. These percentages are matched with the lead-free perovskite construction KCsSnI_1.7_Cl_1.3_. Table S1 is inserted to estimate the elements and their binding energies from the XPS analyses.

**Fig. 2:**
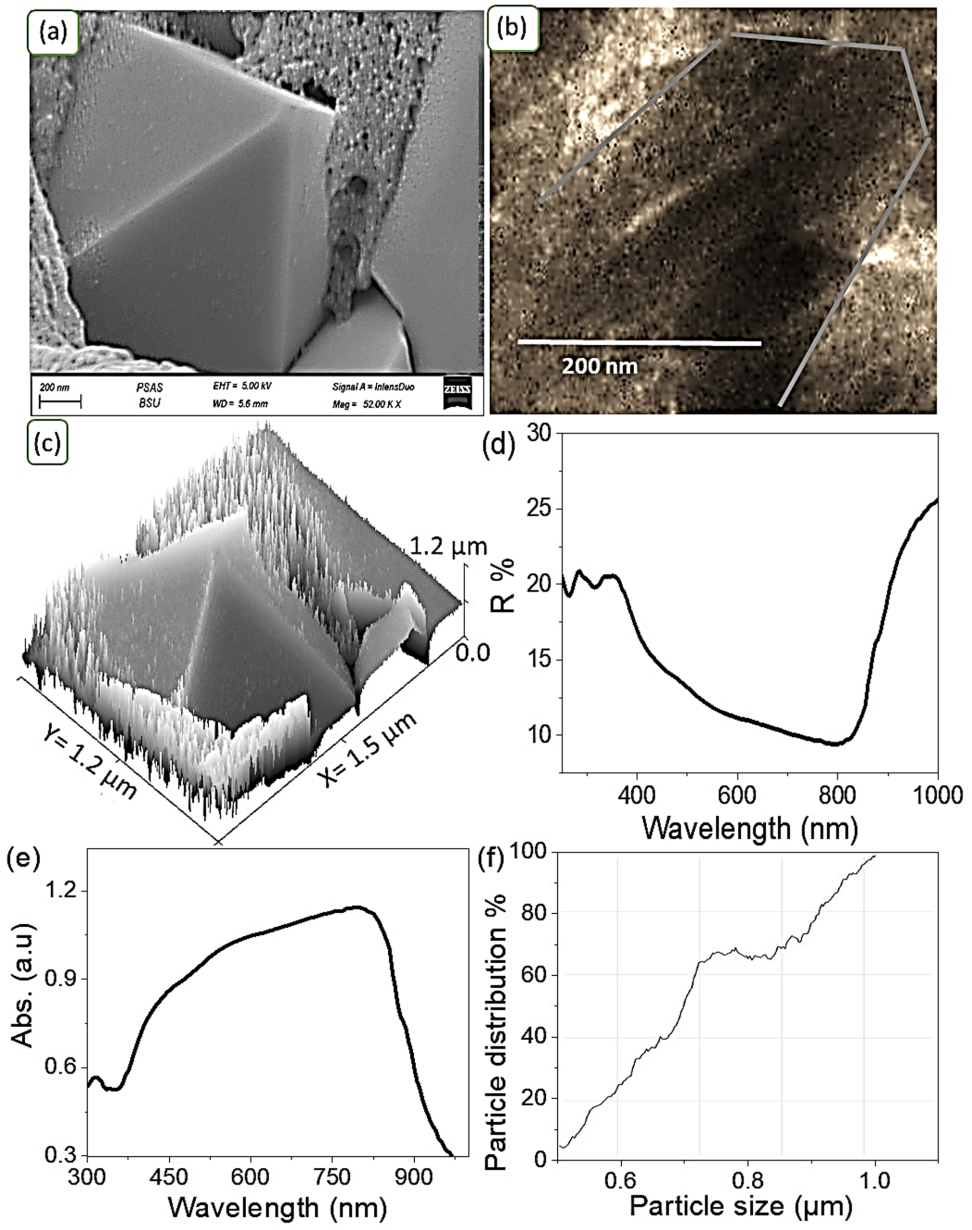
(**a**) SEM image, (**b**) TEM image, (**c**) theoretical modeling of the morphological features, (**d**,**e**) optical reflectance and absorbance spectra, respectively, and (**f**) particle size distribution of the lead-free perovskite KCsSnI_1.7_Cl_1.3_.

The SEM image of the synthesized lead-free perovskite material, KCsSnI_1.7_Cl_1.3_, is presented in Fig. 2a. The material exhibits excellent crystallinity, forming orthorhombic pyramid-shaped crystals without any evidence of pinhole formation. The grain size ranges between 800 and 1000 nm, making this structure highly favorable for optical absorption properties. The significant surface roughness contributes to its ability to capture photons efficiently, which in turn promotes electron generation^31^. The unique orthorhombic, pyramid-like crystal structure of KCsSnI_1.7_Cl_1.3_ contributes significantly to its enhanced optical absorption and photocatalytic performance through several synergistic effects, such as the improvement of light utilization. The pyramidal morphology introduces increased surface irregularity and geometric complexity, which enhances internal light scattering and prolongs the path of photons within the material. This reduces light reflection and boosts photon capture efficiency, thereby strengthening absorption across the visible spectrum, reaching up to 800 nm, as demonstrated in our measurements. Also, this feature provides a larger reactive surface: The faceted architecture of the orthorhombic crystals offers a greater surface area compared to smooth or spherical particles. This expanded interface provides more reactive sites for surface-driven photocatalytic processes, making the material particularly effective for interactions with microbial cells and organic contaminants. In addition to the improvement of the enhanced charge carrier dynamics:

The well-ordered crystal lattice ensures high crystallinity with fewer structural defects, which facilitates faster charge carrier movement and reduces recombination rates of photoinduced electron–hole pairs. This improved charge separation directly translates into higher photocatalytic activity under light exposure.

The TEM analysis, depicted in Fig. 2b, further supports the presence of the orthorhombic shape, confirming the material’s highly crystalline nature at the nanoscale level. This analysis provides additional evidence for the formation of orthorhombic pyramid-shaped crystals in the lead-free perovskite KCsSnI_1.7_Cl_1.3_. The observation of these features at the nanoscale suggests that the suspension of the perovskite material in solution allows for the development of this unique morphology, which is evident in the TEM images.

Additionally, theoretical modeling, as shown in Fig. 2c, corroborates these experimental observations, further confirming the formation of the orthorhombic pyramid shape with a high degree of crystallinity. The model also reveals the presence of smaller particles that help cover any minor imperfections or holes that may form during the crystallization process. This additional layer of particles enhances the material’s structural integrity and supports its optical properties by optimizing photon capture and minimizing defects. Figure 2f illustrates the particle size distribution of the synthesized lead-free perovskite KCsSnI_1.7_Cl_1.3_. The analysis reveals that the majority of the particles fall within the 800 to 1000 nm size range. This well-defined particle size distribution plays a crucial role in determining the optical properties of the material. The relatively large and uniform grain size suggests high crystallinity, which directly impacts the material’s ability to effectively absorb light across a broad spectrum. The reflectance spectrum of the lead-free perovskite material, illustrated in Fig. 2d while the absorbance is in Fig. 2e, indicates a low reflectance up to 800 nm. This low reflectance is a strong indicator of the material’s capacity to absorb photons effectively within this optical range. The efficient photon absorption is critical for generating hot electrons, which contribute to the degradation of organic contaminants, including bacteria, under light exposure. This performance is notably superior compared to its behavior in the dark conditions, highlighting the material’s potential in photocatalytic and optoelectronic applications. The band gap was determined using the Tauc relation (Eq. 2), which involves parameters such as absorbance (A), absorption coefficient (α), Planck’s constant (h), and photon frequency (ν). This analysis is based on the data presented in Fig. 2d, where the calculated band gap is approximately 1.34 eV. This relatively low band gap value indicates strong absorption capabilities in both the visible (Vis) and ultraviolet (UV) regions, making the material highly suitable for optoelectronic applications.

Overall, the combination of SEM, TEM, and theoretical modeling provides a comprehensive understanding of the structural and optical properties of the lead-free perovskite KCsSnI_1.7_Cl_1.3_. The formation of orthorhombic pyramid-shaped crystals with excellent crystallinity, coupled with a high surface roughness and low reflectance, makes this material highly effective for photon capture and electron generation. Its ability to degrade organic matter under light conditions underscores its potential for environmental and optoelectronic applications, especially in areas where light-induced processes are critical for material performance.

2 $$\:{\upalpha\:}\text{h}{\upnu\:}\:=\:\text{A}(\text{h}{\upnu\:}-{E}_{g}{)}^{1/2}$$

### Testing the antimicrobial activity for the synthesized lead-free perovskite KCsSnI_1.7_Cl_1.3_

The antibacterial activity of the lead-free perovskite material KCsSnI1.7Cl1.3 was evaluated using the well diffusion method. The zone of inhibition serves as the primary indicator for measuring the antibacterial efficacy of this perovskite nanomaterial. The tests were conducted at three different concentrations—100, 200, and 400 ppm—against both Gram-positive bacteria (*Bacillus subtilis* and *Staphylococcus aureus*) and Gram-negative bacteria (*Escherichia coli* and *Salmonella species*). These experiments were carried out under standard conditions, maintaining a pH of 7 and a temperature of 37 °C on Mueller-Hinton Agar (MHA) plates. The supernatant from each bacterial culture was used as the reference control (R).

**Fig. 3:**
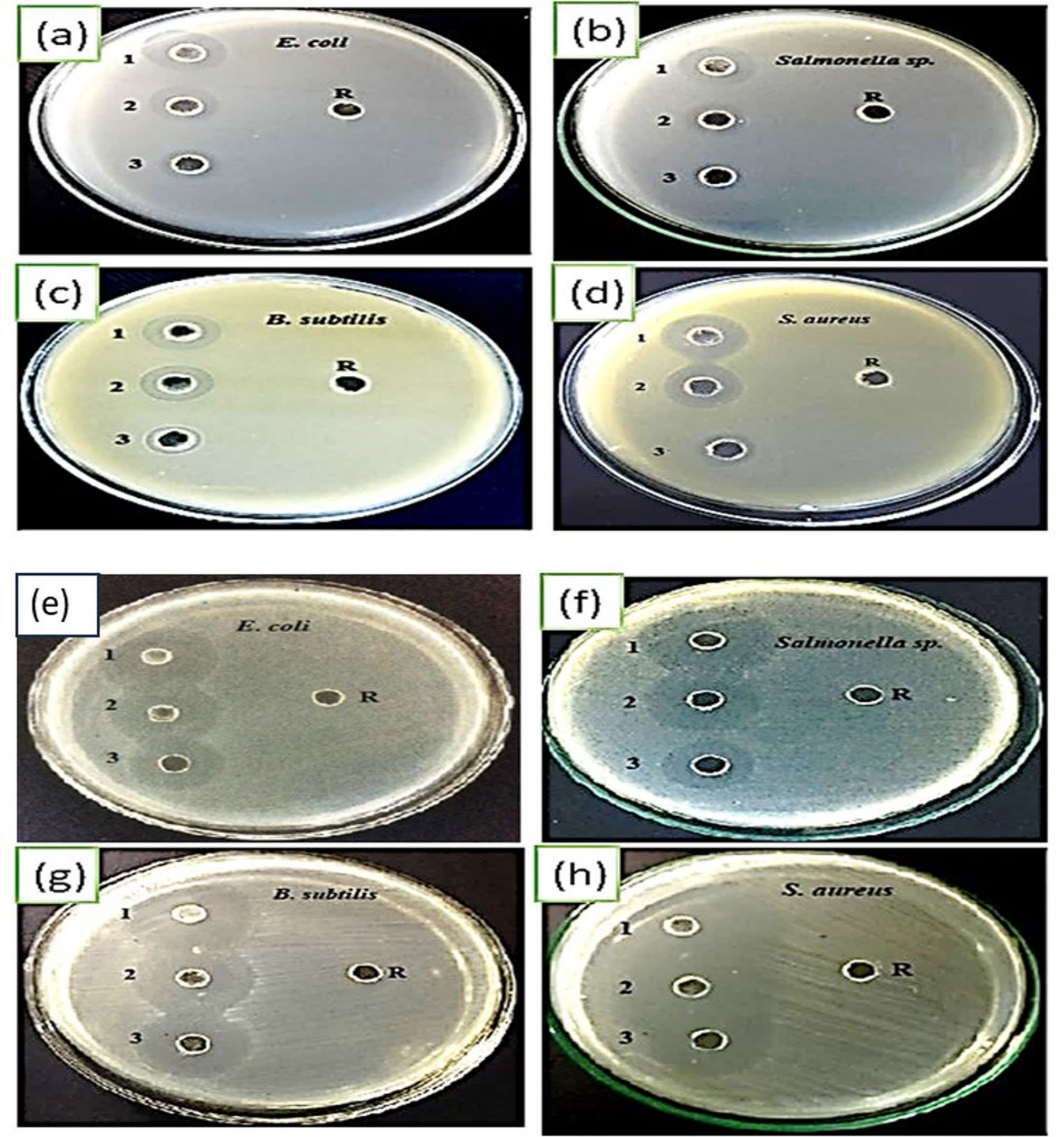
Antibacterial activity assays at varying concentrations (100, 200, and 400 ppm) of KCsSnI_1.7_Cl_1.3_ perovskite against the tested pathogens, conducted under dark conditions (Group A) and UV light exposure (Group B).

The results, displayed in Fig. 3; Table 1, demonstrate that the lead-free perovskite KCsSnI1.7Cl1.3 material exhibits broad-spectrum antibacterial activity against all tested bacterial strains. Table S2 represents the KCsSnI_1.7_Cl_1.3_ material, which exhibited broad-spectrum antibacterial activity against all tested bacterial strains, allowing for repeating second runs to confirm reproducibility. Among them, the highest zone of inhibition was observed against Staphylococcus aureus, with measurements of 17 mm in the dark and 35 mm under UV light. The order of bacterial inhibition was *S. aureus* > *B. subtilis* >* E. coli *> *Salmonella* sp. Notably, the inhibition zones increased when the nanomaterial was exposed to UV light, indicating that the perovskite nanoparticles possess significant photocatalytic properties. Additionally, the zones of inhibition expanded as the concentration of the nanomaterial increased from 100 to 400 ppm. No inhibition was observed in the control wells containing only the supernatants from the bacterial cultures.

The antimicrobial effect of the perovskite material in the dark can be attributed to electrostatic interactions between the positively and negatively charged components of the perovskite and the bacterial cells. The perovskite nanomaterial’s double charges play a crucial role in its antibacterial activity. For instance, the cesium (Cs) and tin (Sn) ions, which carry positive charges, are particularly effective in interacting with Gram-negative bacteria, while the negatively charged iodine (I) and chloride (Cl) ions target Gram-positive bacteria. These electrostatic attractions disrupt the bacterial cell membrane, making it more permeable and ultimately leading to the degradation of the bacterial cell structure.

Under light conditions, the antibacterial activity of KCsSnI_1.7_Cl_1.3_ is enhanced due to a dual mechanism involving both electrostatic attraction and photocatalytic degradation. The material’s strong light absorption capabilities, a result of its narrow bandgap, enable it to capture photons at its active sites. This photon absorption generates hot electrons and positive holes. In Gram-positive bacteria, the hot electrons are transferred, leading to bacterial degradation through the generation of hydroxyl (OH.) radicals. In contrast, Gram-negative bacteria absorb the positive holes, resulting in the formation of hydrogen (H.) radicals, which cause the breakdown of the bacterial cell membrane and eventually the entire cell^32,33^.

**Fig. 4:**
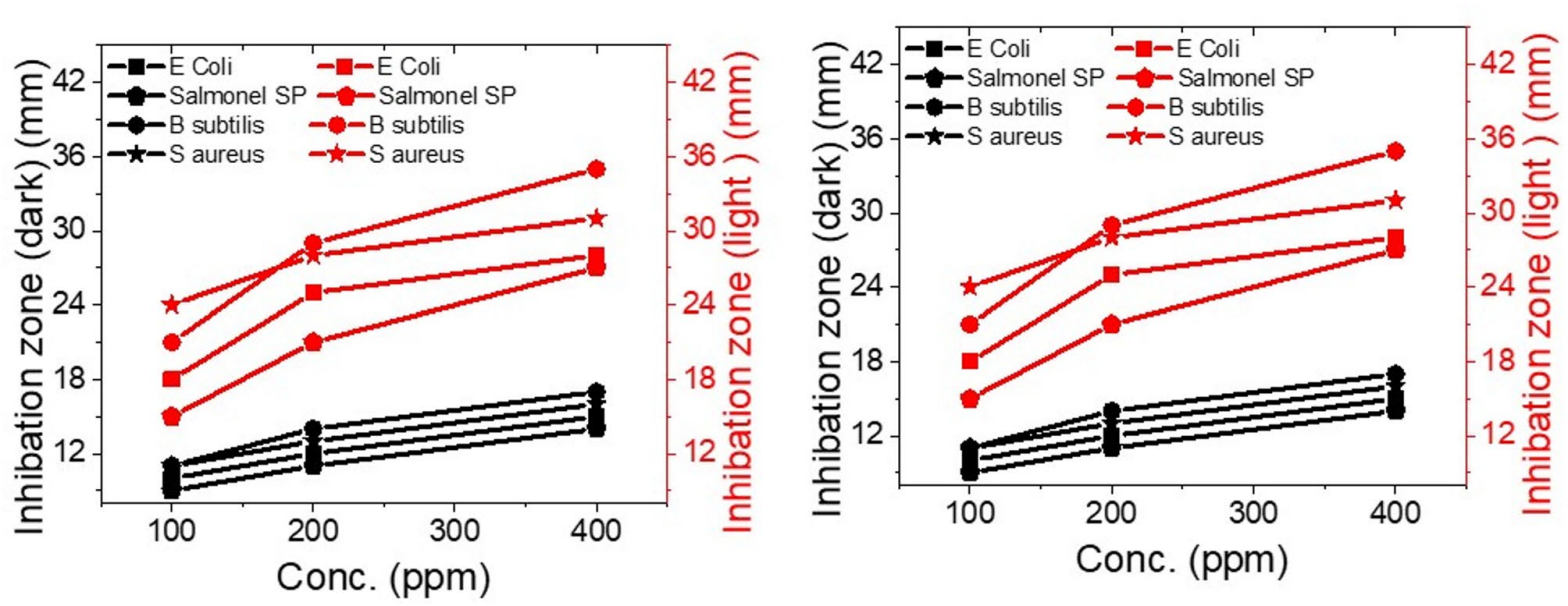
Antimicrobial activity of the lead-free perovskite KCsSnI_1.7_Cl_1.3_ material against *E. coli*, *Salmonella* sp., *B. subtilis*, and *S. aureus* under dark conditions (black curve) and light exposure (red curve).

The differences in photocatalytic activity between light and dark conditions are clearly depicted in Fig. 4. The red curves represent the enhanced antibacterial performance of the perovskite material under light exposure, while the black curves illustrate its activity in the dark. These results underscore the significant role of light in improving the antibacterial effectiveness of the lead-free perovskite material.

So, the lead-free perovskite KCsSnI_1.7_Cl_1.3_ exhibits robust antibacterial properties through electrostatic attraction in the dark conditions and additional photocatalytic degradation under light. The material’s effectiveness against both Gram-positive and Gram-negative bacteria, coupled with its ability to increase antibacterial activity under UV light, highlights its potential for applications in antibacterial treatments and related fields.

**Table 1:** Antibacterial activity of lead-free perovskite KCsSnI_1.7_Cl_1.3_ at various concentrations (100, 200, and 400 ppm) at 37 °C, both in the dark (black numerals) and under UV light (red numerals). The table presents the corresponding Inhibition zones for Gram-negative and Gram-positive bacteria.

Observation no.	Concentration of nanomaterial (ppm)	Diameter of the inhibition zones (mm) (In dark/under UV)			
		Bacteria			
		Gram (−)		Gram (+)	
		*E. coli*	*Salmonel* sp.	*B. subtilis*	*S. aureus*
1	400	15/28	14/27	17/35	16/31
2	200	12/25	11/21	14/29	13/28
3	100	10/18	9/15	11/21	11/24

The antibacterial mechanism of the lead-free halide perovskite KCsSnI_1.7_Cl_1.3_ is based on a dual-action strategy: photocatalytic activity under UV illumination and electrostatic interactions with bacterial cell walls. This mechanism provides broad-spectrum antibacterial efficacy against both Gram-positive (e.g., *Staphylococcus aureus*, *Bacillus subtilis*) and Gram-negative (e.g., *Escherichia coli*, *Salmonella sp.*) bacteria.

Upon exposure to UV light, KCsSnI_1.7_Cl_1.3_ exhibits strong optical absorption, which excites electrons from the valence band to the conduction band, generating electron-hole pairs (e^−^/h^+^). These charge carriers interact with water and dissolved oxygen in the medium, initiating a cascade of photocatalytic reactions. The excited electrons (e^−^) reduce molecular oxygen (O_2_) to produce superoxide radicals (O_2_^−·^), while the photogenerated holes (h^+^) oxidize water molecules or hydroxide ions to generate hydroxyl radicals (·OH). Both radical species are potent Reactive Oxygen Species (ROS) known for causing oxidative stress in bacterial cells.

The major ROS involved in the antibacterial process are: Hydroxyl radicals (·OH): These highly reactive species damage lipid membranes, proteins, and nucleic acids. Superoxide radicals (O_2_^−·^): These contribute to protein oxidation and interfere with cellular respiration. Hydrogen peroxide (H_2_O_2_): Formed through subsequent reactions of O_2_^−·^, H_2_O_2_ can penetrate cells and cause additional oxidative damage.

This photocatalytic ROS generation is responsible for the degradation of bacterial cell walls, leading to leakage of cytoplasmic contents and eventual cell death. The enhancement in the inhibition zone under UV irradiation (compared to dark conditions) supports this photocatalytic action, as demonstrated in Table 1; Figs. 3 and 4. To further support this mechanism, future validation through ROS quantification assays (e.g., DCFH-DA) and scavenger studies (using isopropanol, EDTA, or benzoquinone as radical quenchers) is planned, as recommended. In parallel, the antibacterial action is also supported by electrostatic interactions. The perovskite material exhibits surface charge polarity, which facilitates strong attraction with oppositely charged bacterial membrane components. Gram-positive bacteria possess thick peptidoglycan layers embedded with negatively charged teichoic acids, whereas Gram-negative bacteria feature lipopolysaccharides on their outer membranes that also carry negative charges. The positive surface sites of the perovskite interact with these negative components, leading to membrane destabilization and increased permeability.

**Fig. 5:**
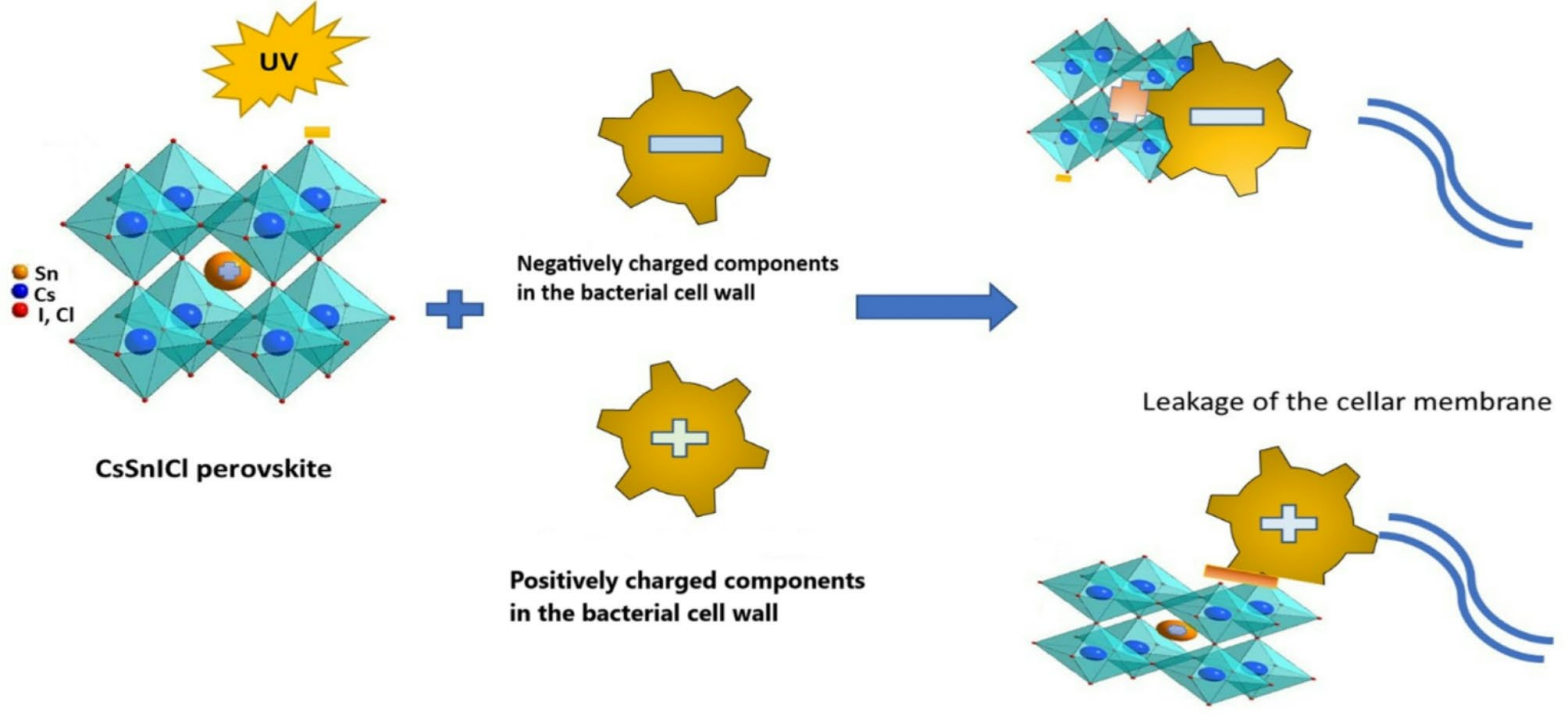
The mechanism of the antibacterial activity using the lead-free perovskite KCsSnI_1.7_Cl_1.3_ materials.

The mechanism illustrated in the updated schematic (see revised Fig. 5) reflects both contributions: Electrostatic adsorption and interaction, resulting in physical damage to the bacterial envelope. Photocatalytic ROS-mediated degradation of intracellular and membrane components under UV light. This synergistic antibacterial mechanism explains the high efficacy of KCsSnI_1.7_Cl_1.3_ nanomaterials against both bacterial classes. Gram-negative bacteria are more resistant due to their outer membrane, but the strong photocatalytic oxidation and electrostatic disruption together overcome this barrier.

## Conclusions

The lead-free perovskite KCsSnI_1.7_Cl_1.3_ synthesized in this study exhibits a unique combination of excellent crystallinity, broad optical absorption, and potent antimicrobial activity, positioning it as a multifunctional material for sustainable applications. KCsSnI_1.7_Cl_1.3_ offers enhanced advantages due to its dual-cation structure (K^+^ and Cs^+^) that stabilizes the Sn^2+^ oxidation state and maintains high structural integrity under ambient conditions. Its orthorhombic, pyramid-shaped crystals and strong light absorption up to 800 nm make it particularly suitable for light-driven applications. Importantly, KCsSnI_1.7_Cl_1.3_ demonstrates strong antibacterial activity against both Gram-positive and Gram-negative bacteria, which is significantly amplified under UV illumination. This effect is attributed to a dual mechanism involving electrostatic membrane disruption and photocatalytic ROS generation. Such broad-spectrum and light-enhanced antibacterial performance distinguishes it from other lead-free perovskites that typically lack this dual functionality. Moreover, the material’s eco-friendly composition, low synthesis temperature, and short processing time support its scalability for industrial use. As such, KCsSnI_1.7_Cl_1.3_ holds significant promise for integration into practical applications such as water disinfection, antimicrobial coatings, and environmental remediation systems. Future studies will focus on assessing the long-term stability, reusability, and performance of this material under diverse environmental conditions, including real wastewater and biological systems. Expanding its antimicrobial testing to include viruses and resistant strains, as well as incorporating it into composite films and membrane systems, could further enhance its applicability. Overall, KCsSnI_1.7_Cl_1.3_ represents a next-generation lead-free perovskite with superior multifunctionality, offering valuable contributions to sustainable materials science and public health technologies.

## Supplementary Information

Below is the link to the electronic supplementary material.

Supplementary Material 1

## Data Availability

The datasets (Tables and Figures) generated and analyzed during the current study are available from the corresponding author upon reasonable request.
