# Supplementary material for: Lead-free perovskite KCsSnI1.7Cl1.3 material exhibiting superior photocatalytic antimicrobial activity
Source: Sci Rep. 2025 Sep 1;15:32185. doi: 10.1038/s41598-025-17357-8 (PMC12402134; doi:10.1038/s41598-025-17357-8)
Supplement: Supplementary file 1 — Supplementary Material 1 [file 41598_2025_17357_MOESM1_ESM.docx]

**Lead-Free Perovskite KCsSnI_1.7_Cl_1.3_** **Material Exhibiting Superior Photocatalytic Antimicrobial Activity**

**Ahmed M. Mahmoud^1^, Canan Can^2^, Mohamed Rabia^3^**

^1^Department of Botany and Microbiology, Faculty of Science, Beni-Suef University, Beni-Suef 62511, Egypt; [ahmed.mahmoud@science.bsu.edu.eg](mailto:ahmed.mahmoud@science.bsu.edu.eg)

^2^Faculty of Arts and Sciences, Department of Biology, Division of Molecular Biology, Gaziantep University, Gaziantep, Turkey; [can@gantep.edu.tr](mailto:can@gantep.edu.tr)

^3^Nanomaterials Science Research Laboratory, Chemistry Department, Faculty of Science, Beni-Suef University, Beni-Suef, Egypt; [mohamedchem@science.bsu.edu.eg](mailto:mohamedchem@science.bsu.edu.eg)

Corresponding author: Ahmed M. Mahmoud [ahmed.mahmoud@science.bsu.edu.eg](mailto:ahmed.mahmoud@science.bsu.edu.eg)

Table S1. XPS analysis showing the elemental composition and corresponding binding energies of the detected elements.

| Element | Transition | Binding energy (eV) |
| --- | --- | --- |
| Cl | 2p | 202.7 |
| K | 2p | 296.4 |
| Sn | 3d | 487.2 |
| I | 3d | 620.1 |
| Cs | 3d | 718.8 |
| C | 1s | 287.1 |
| N | 1s | 404.0 |
| O | 1s | 534.8 |

**Table S2.** Antibacterial activity of lead-free perovskite KCsSnI_1.7_Cl_1.3_ at various concentrations (100, 200, and 400 ppm) at 37 °C, both in the dark (black numerals) and under UV light (red numerals). The table presents the corresponding inhibition zones for Gram-negative and Gram-positive bacteria (the repeating second runs)**.**

| **Diameter of the inhibition zones (mm)**  **(In dark/ under UV)** | | | | **Concentration of nanomaterial (ppm)** | **Observation No.** |
| --- | --- | --- | --- | --- | --- |
| **Bacteria** | | | |  |  |
| **Gram (+)** | | **Gram (-)** | |  |  |
| *S. aureus* | *B. subtilis* | *Salmonel SP* | E coli |  |  |
| 16/29  13/30  10/24 | 16/35  15/27  11/20 | 13/27  11/23  9/17 | 16/28  11/27  10/20 | 400  200  100 | 1  2  3 |
